# *Mycobacterium tuberculosis* Phosphoribosyltransferase Promotes Bacterial Survival in Macrophages by Inducing Histone Hypermethylation in Autophagy-Related Genes

**DOI:** 10.3389/fcimb.2021.676456

**Published:** 2021-07-26

**Authors:** Srabasti Sengupta, Barsa Nayak, Michael Meuli, Peter Sander, Snehasish Mishra, Avinash Sonawane

**Affiliations:** ^1^ School of Biotechnology, KIIT Deemed to be University, Bhubaneswar, India; ^2^ Department of Biosciences and Biomedical Engineering, Indian Institute of Technology Indore, Indore, India; ^3^ Institut für Medizinische Mikrobiologie, Universität Zürich, Zurich, Switzerland; ^4^ Nationales Zentrum für Mykobakterien, Zürich, Switzerland

**Keywords:** Tuberculosis, *Mycobacterium tuberculosis*, Autophagy, Histone hypermethylation, MAPK pathway, epigenetic modification

## Abstract

*Mycobacterium tuberculosis (Mtb)* inhibits autophagy to promote its survival in host cells. However, the molecular mechanisms by which *Mtb* inhibits autophagy are poorly understood. Here, we report a previously unknown mechanism in which *Mtb* phosphoribosyltransferase (*Mtb*PRT) inhibits autophagy in an mTOR, negative regulator of autophagy, independent manner by inducing histone hypermethylation (H3K9me2/3) at the *Atg5* and *Atg7* promoters by activating p38-MAPK- and EHMT2 methyltransferase-dependent signaling pathways. Additionally, we find that *Mtb*PRT induces EZH2 methyltransferase-dependent H3K27me3 hypermethylation and reduces histone acetylation modifications (H3K9ac and H3K27ac) by upregulating histone deacetylase 3 to inhibit autophagy. In summary, this is the first demonstration that *Mtb* inhibits autophagy by inducing histone hypermethylation in autophagy-related genes to promote intracellular bacterial survival.

## Introduction

Pathogens are equipped with various strategies to dampen the host immune responses. Upon infection, a battle between the host and the pathogen occurs, wherein the pathogen strives to command the host defence, and the host endeavours to eliminate the pathogen. This orchestrated tussle involves alterations in cell-signalling cascades and genome regulatory mechanisms. Accumulating evidence demonstrates that pathogens can reprogram host gene expression to facilitate their survival by inducing various histone modifications such as methylation, acetylation, and phosphorylation that control the accessibility of activation or repression transcription factors to target genes (Hamon and Cossart, 2008; Allis and Jenuwein, 2016). For example, *Anapalsma phagocytophilum* transcriptionally silences host defence genes by inducing histone deacetylation (Cabezas-Cruz et al., 2016), *Chlamydia trachomatis* promotes histone methylation (Pennini et al., 2010), *Escherischia coli* induces DNA methylation to down-regulate the tumor suppressor CDKN (Tolg et al., 2011), and *Shigella flexneri* infection inhibits MAPK-dependent histone H3 serine 10 (H3S10) phosphorylation to impair the recruitment of nuclear factor-kappa B (NF-κB) at the interleukin-8 (IL-8) promoter (Philpott et al., 2000). Moreover, some bacterial proteins interact with host chromatins to modulate transcription of genes involved in host defence mechanisms (Rolando et al., 2013; Yaseen et al., 2015). Examples include *Listeria monocytogens* listeriolysin O which dephosphorylates H3 and deacetylates H4 to suppress host immunity factors (Hamon et al., 2007) and *Mycobacterium tuberculosis* (*Mtb*) ESAT-6 and LpqH proteins induce histone modifications into the MHC class II transactivator promoter to inhibit MHC-II expression and antigen presentation (Pennini et al., 2006; Kumar et al., 2012). Similarly, *Mtb* Rv1988 hyper-methylates histone H3 to repress host genes involved in the defence against mycobacteria (Yaseen et al., 2015). Another report showed histone methyl transferase SET8 induces H4K20me1 to regulate apoptosis and inflammation to assist *Mtb* survival (Singh et al., 2017). Then, (Chandran et al., 2015) showed that *Mtb* suppresses IL-12B expression *via* HDAC1. Thus, there is considerable evidence that epigenetic modifications are critical determinants of bacterial virulence.

*Mtb*, which causes human tuberculosis (TB), is one of the most successful and devastating pathogens (Glickman and Jacobs, 2001). This is because *Mtb* is profoundly evolved with plethora of strategies to weaken host immunity. Such strategies include inhibition of phago-lysosome fusion, oxidative stress, antigen presentation, and T-cell immunity (Forrellad et al., 2013). Inhibition of the host’s autophagy machinery is another predominant mechanism by which *Mtb* increases intracellular persistence (Deretic et al., 2006; Mohanty et al., 2015). Autophagy involves the synthesis of a double-membrane structure known as the autophagosome, which sequesters cytoplasmic proteins and organelles. These matured autophagosomes then subsequently fuse with acidified lysosomes to degrade their contents (Ohsumi, 2014). This process involves a series of dynamic membrane rearrangements by a set of autophagy-related (ATG) proteins (Mizushima et al., 2011; Nishimura and Tooze, 2020). ATG5 and ATG7 are crucial autophagy-inducing molecules and LC3 is an autophagy indicator (Arakawa et al., 2017). *Mtb* has developed extraordinary attributes to evade autophagy-dependent immune surveillance mechanisms. Induction of autophagy in infected macrophages targeted *Mtb* to lysosomal degradation, thus reducing its intracellular survival (Gutierrez et al., 2004). A genome-wide screen identified 44 autophagy-related genes responsible for the *Mtb* clearance (Kumar et al., 2010). Studies in autophagy-deficient mice also confirmed that autophagy protects against active TB by decreasing bacterial burden and inflammation (Castillo et al., 2012).

Emerging data suggest that p38 mitogen activated protein (p38-MAP) kinase, which links signal transduction molecules during biological processes (Zarubin and Han, 2005), is involved in the inhibition of autophagy. Blockade of p38 MAPK increases autophagy by facilitating the interaction between p38 interacting protein (p38IP) and autophagy protein 9 (ATG9) (Henson et al., 2014). Another report showed that p38 MAPK phosphorylates the autophagy inducer ULK1 protein to inhibit autophagy (He et al., 2018). Despite increasing awareness of the importance of the transcriptional regulation of autophagy during stress conditions, epigenetic control of bacterial infections is a largely unexplored phenomenon. Few reports have demonstrated that histone modifications regulate autophagy to determine cellular fate (Füllgrabe et al., 2014; Lapierre et al., 2015; Byun et al., 2020).

Several studies have clearly demonstrated that *Mtb* inhibits autophagy to promote its intracellular persistence; however, the underlying molecular mechanisms of autophagy inhibition during *Mtb* infection are poorly understood. Our previous study showed that the *Mtb* phosphoribosyltransferase (*Mtb* PRT) enzyme, found in the cell wall of *Mtb*, inhibits autophagy to facilitate *Mtb* persistence inside macrophages and zebrafish (Mohanty et al., 2015). Here, we report for the first time that *Mtb* inhibits autophagy by two concurrent mechanisms; inducing histone methylation enrichment that causes transcriptional repression, and down-regulating histone acetylations that cause transcriptional activation in autophagy-related genes. We report that *Mtb* PRT inhibits autophagy in an mTOR-independent manner by inducing histone H3 lysine 9 (H3K9me2/3) and lysine 27(H3K27me3) hypermethylation at the promoter regions of *Atg5* and *Atg7* genes involved in p38 MAPK-, EHMT2- and EZH2 methyltransferase-dependent signalling pathways. We further show that *Mtb* PRT reduces transcriptional activation H3K9ac and H3K27ac histone modifications by upregulating of HDAC3 (histone deacetylase 3) expression to inhibit autophagy. To the best of our knowledge, this is the first report to demonstrate that *Mtb* introduces both transcriptional activation and repression of epigenetic modifications to inhibit autophagy and aids its cellular persistence.

## Materials and Methods

### Ethical Statement

All experiments were approved by the Institutional Biosafety committee of KIIT University (vide DBT memorandum No-BT/BS/17/493/2012-PID). All the bacterial mutants were handled in adherence to experimental guidelines and procedures approved by the Institutional Biosafety Committee (IBSC) of School of Biotechnology, KIIT University (KIIT/3-12). All studies involving virulent mycobacterial strains were carried out at the BSL-3 facility at Universität Zürich, Zurich (Switzerland). Animal care and use protocol adhered were approved by national guidelines of the Committee for the Purpose of Control and Supervision of Experiments on Animals (CPCSEA), Government of India.

### Chemicals, Reagents and Cell Culture Conditions

*Mycobacterium smegmatis* mc^2^155 was grown in Middlebrook’s 7H9 broth medium (Difco, New Jersey, USA) containing 0.05% Tween 80, 0.5% glucose and 0.5% albumin at 37°C on a shaker at 120 rpm. Murine RAW264.7 macrophage cell line was cultured in Dulbecco’s Modified Eagle’s medium (DMEM; HiMedia, Mumbai, India) supplemented with 10% fetal bovine serum, 1% penicillin-streptomycin solution, and 1% L-glutamine. The cells were seeded onto 24-well and 6-well culture dishes at a density of 2x10^5^ cells/ml and 1x10^7^ cells/ml, respectively and proceeded for experiments. Anti- ATG5, anti-ATG7, anti-Beclin1, anti- H3K9me3, anti- H3K27me3, anti- H3K9ac, anti- H3K27ac, anti-HDAC1, anti-HDAC2, anti-HDAC3, anti-phospho–p38, anti-p62/SQSTM1, anti-GAPDH, anti-β-actin, and secondary goat anti-rabbit and goat anti-mouse antibodies were purchased from Cell Signaling Technologies (Massachusetts, USA). Anti-LC3I/II antibody was purchased from Sigma (Missouri, USA). All the pharmacological inhibitors were purchased from Sigma (Missouri, USA) and Calbiochem (Massachusetts, USA) and reconstituted in DMSO (Himedia, Mumbai, India) or sterile H_2_O at the following concentrations: U0126 (10 µM), SB203580 (10 µM), UNC0638 hydrate (5µM), rapamycin (50nM) and 3MA (10mM).

### Construction of *M. tuberculosis* Phosphoribosyltransferase Deletion Mutant

A 1.5 kb fragment comprised of the upstream region of *Rv3242c* and 129 bp of the 5’ part of *Rv3242c* was amplified with primers (CA’TATGGGTAGTCGTTGACGGTGACG; forward) and (GTT’AACGAGTCGGTCCGGGTCTTG; reverse) containing *NdeI* and *HpaI* restriction sites using *Mtb* H37Rv genomic DNA as a template. Likewise, a 1.4 kbp fragment comprisingof 69 bp of the 3’ part of *Rv3242c* and its downstream region was amplified with primers (GTT’AACGTCAACACGAGGACTCACCA, forward and A’CATGTCCAGTTCGCCC TGACCTA, reverse) containing *Hpa* and *PscI* restriction sites. The fragments were initially cloned into pGEM-T Easy vector (Promega, Madison, USA) and transformed into *E. coli* XL1-blue. Recombinant *E. coli* strains were propagated and fragments were isolated from plasmids by restriction enzyme digestion and gel purification and were stepwise cloned into the suicide vector pMCS5-rpsL-hyg, containing a hygromycin resistance cassette for positive selection and an *rpsL* ^+^ allele for counter-selection in a mycobacteria strain with a*rpsL* mutation conferring streptomycin resistance (Brülle et al., 2013) to result in pMCS5-rpsL-hyg-ΔRv3242c. The plasmid was transformed into electrocompetent *Mtb* SMR strain (Davis et al., 2002). The transformants were selected on 7H10 agar plates containing hygromycin (25 mg/L). Single crossover transformants resulting from intermolecular homologous recombination between the suicide vector and the *Rv3242c* genomic locus were identified by Southern blot analysis and subsequently subjected to counter selection on 7H10 plates containing streptomycin (100 mg/L). Deletion mutant (*MtbΔPrt*) with a 444 bp in-frame deletion in *Rv3242c* were identified by Southern blot analysis of a genomic DNA digested with *AgeI* and hybridized to a 128 bp *Rv3242c* 5’ probe amplified with primers (CGTGCGGTTCACCGGC, forward) and (TGACCGCGACACTTGGTGTG, reverse) using genomic DNA as a template.

### Western Blot Analysis

RAW264.7 cells were infected with mycobacterial strains. After 24 h of infection, protein samples were prepared by cell lysis using RIPA buffer (HiMedia, Mumbai, India) containing 5mM EDTA, 5mM EGTA, 1 mM PMSF, protease inhibitor cocktail, 50 mM NaF, 1mM DTT and 1mM Sodium orthovanadate. Proteins were electrophoresed in 12% SDS-PAGE and transferred to polyvinylidene difluoride membrane (PVDF) (GE Healthcare Life sciences) overnight at 28 volts. Blots were blocked with 5% BSA or skimmed milk in TBST (20 mM Tris-HCl, pH 7.4, 137 mM NaCl and 0.1% Tween 20) for 60 min. Then the blots were incubated with primary rabbit IgG antibodies (1:1000) overnight at 4°C and then with HRP-conjugated anti-rabbit or anti-mouse IgG secondary antibodies in 5% BSA or skimmed milk (1:1000) for 2 h. The membrane was washed using 1X TBST and X-ray film was developed using standard chemiluminescent solvent. β-actin and GAPDH were used as loading controls. Each desired protein band densities were quantified by ImageJ software with respect to their corresponding loading controls. For LC3, ratio of LC3 I to II (LC3II/LC3I) with respect to corresponding loading control were calculated and plotted onto graphs for representation.

Similarly, mice bone marrow derived macrophages (BMDMs) (8x10^5^ cells) were infected with mycobacterial strains followed by UNCO638 inhibition for western blot analysis.

### Confocal Microscopy

RAW 264.7 macrophages (5X10^4^) were seeded on coverslips. After infection, the cells were treated with UNCO638, washed with 1XPBS and fixed with 4% PFA followed by incubation for 30 min at 37°C. Then the cells were permeabilized with blocking agent (5% BSA and 0.1% saponin). The cells were then incubated overnight with anti-LC3 antibody (1:250, Sigma, Missouri, USA) at 4°C and then stained with secondary antibodies for 2 h at room temperature. Finally, the cells were mounted in mounting solution with DAPI and the images were analysed using LEICA laser scanning confocal microscope.

### RNA Isolation and Quantitative Real-Time RT-PCR

Total RNA was isolated from the infected or uninfected macrophages using TRIzol reagent (Invitrogen, California, USA) as per the manufacturer’s protocol. cDNA synthesis kit (Thermofisher Scientific, Massachusetts, USA) was used for reverse transcription according to the manufacturer’s protocol. Quantitative real time RT-PCR amplification was performed for quantification of target gene expression using SYBR Green PCR mixture (KAPA Biosystems) in Realplex master cycler (Eppendorf, Hamburg, Germany) with initial denaturation at 95°C for 10 min, final denaturation at 95°C for 30 s, annealing at 52°C for 30 s and extension at 72°C for 30 s to generate 200-bp amplicons. All reactions were repeated at least thrice independently to ensure reproducibility of the result. The mRNA levels were normalized to the transcript levels of *gapdh* and the relative fold changes were calculated.

### Chromatin Immunoprecipitation (ChIP) Assay

For ChIP assay, RAW 264.7 (1X10^7^) cells were seeded onto 100 mm tissue culture disks and infected with mycobacterial strains. After 24 h of infection, cells were washed twice with 1X PBS and then crosslinked with 11% formaldehyde solution for 15 min followed by 2.5 M glycine treatment for quenching formaldehyde solution. The cells were washed with ice cold 1X PBS twice. The cells were then harvested by scrapping using ice cold 1X PBS and centrifuged at 2500 rpm for 5 min at 4°C followed by washing with 1X PBS. The pellets were resuspended in ice-cold 1ml Farnham buffer and centrifuged at 2000 rpm for 5 min at 4°C. The pellet was resuspended with 300 µl of RIPA buffer and kept on ice for 10 min followed by sonication in Bioruptor at high setting for a total time of 40 min (30 seconds ON and 30 seconds OFF) at 4°C. The chromatin length was verified and proceeded for further steps. The sonicated mixture was centrifuged at 14000 rpm for 15 min at 4°C. The supernatant was collected, quantified and adjusted to volume with RIPA buffer so that each reaction contains 150 µg/ml of chromatin. The suspension was incubated with previously prepared Protein-A sepharose beads for 1h at 4°C in a rotator. After centrifugation at 1500 rpm for 2 min at 4°C, the supernatant was taken and incubated overnight with 6 µg of antibodies against H3K9me2/3 and H3K27me3 per IP in rotator at 4°C. Next day, the suspensions were again incubated with Protein-A sepharose beads for 2 h in rotator at 4°C and centrifuged at 2000 rpm for 1 min. The pellets were washed using LiCl wash buffer (7-8times) and TE buffer (once). The pellet was dissolved in IP elution buffer for 30 min at RT and the supernatants were left at 65°C overnight for reverse cross-linking. Next day, RNA and protein were digested with RNase and Proteinase K to obtain purified DNA. Isolated DNA was further processed for qPCR using specific primers for *Atg5, Atg7* and *gapdh*promoters. The qPCR data were normalized to input DNA. Primers for *gapdh* promoter were used as a negative control.

### Macrophage Infection Assay

*Msm* harbouring pSMT3 plasmid *(Msm_pSMT3_*) and recombinant *Msm* expressing *Mtb PRT (Rv3242c)* (*Mtb_Prt_*) strains were grown to mid-exponential phase. Bacterial cultures were pelleted, washed in 1X PBS and re-suspended in DMEM medium to a final OD_600_ 0.1. Bacterial clumps were broken by ultrasonication for 5 min followed by a low-speed centrifugation for 2 min. RAW264.7 macrophages (2x10^5^ cells/well) were seeded on 24-well tissue culture plates with media containing no antibiotic solution and grown for 18-20 h. The cells were infected at a multiplicity of infection (MOI) 10, treated with UNCO638 (5µm) and intracellular bacterial survival was determined by lysis of infected macrophages with 0.5% Triton-X 100 at different time points and plating the serially diluted samples onto 7H9 plates. The equal input and time zero (T_0_) count of infecting bacilli were determined to calculate the percentage survival (% survival= CFU at specific time/CFU of bacteria added for infection X 100).

To determine the intracellular survival of *Mtb* and *MtbΔPrt*, RAW 264.7 (2.5 X 10^5^) cells were seeded onto 48-well plate and infected at a MOI of 1. After 0, 3 and 5 days of infection, the adherent cells were covered with ice cold dH_2_O for 10 min at RT followed by further incubation with 7H9 media and 0.17% SDS. The pellets were resuspended, plated onto 7H10 plates and incubated at 37°C followed for CFU enumeration.

### Isolation of BMDM

Six to eight weeks old Balb/C mice were sacrificed by cervical dislocation. The femur and tibia bones were flushed with RPMI by inserting a 26-gauge needle. The marrow was dispersed by passing through a 19-gauge needle twice. The isolated cells were passed through a 70 µm cell strainer (Himedia, Mumbai, India). The strained cells were centrifuged at 1500 rpm for 5 min at 4°C and the cell pellet was washed with 1X RBC lysis buffer (Sigma, Missouri, USA) to remove the contaminating RBCs. The cells were briefly centrifuged, washed and counted after staining with trypan blue (Sigma, Missouri, USA) counterstain. Appropriate number of cells were seeded onto 6 well plate in presence of 20 ng/ml recombinant macrophage colony stimulating factor (M-CSF) for 7 days and then used for infection assays.

### Statistical Analysis

All experiments were performed at least three times (n=3). Statistical analyses were performed using the Mann-Whitney U-test (two-tailed, equal variances). Significance was referred as: *** for P<0.001, ** for P ≤ 0.01 and * for P ≤ 0.05.

## Results

### *M. tuberculosis* Phosphoribosyltransferase Inhibits Autophagy Through an mTOR-Independent Mechanism

**Figure 1:**
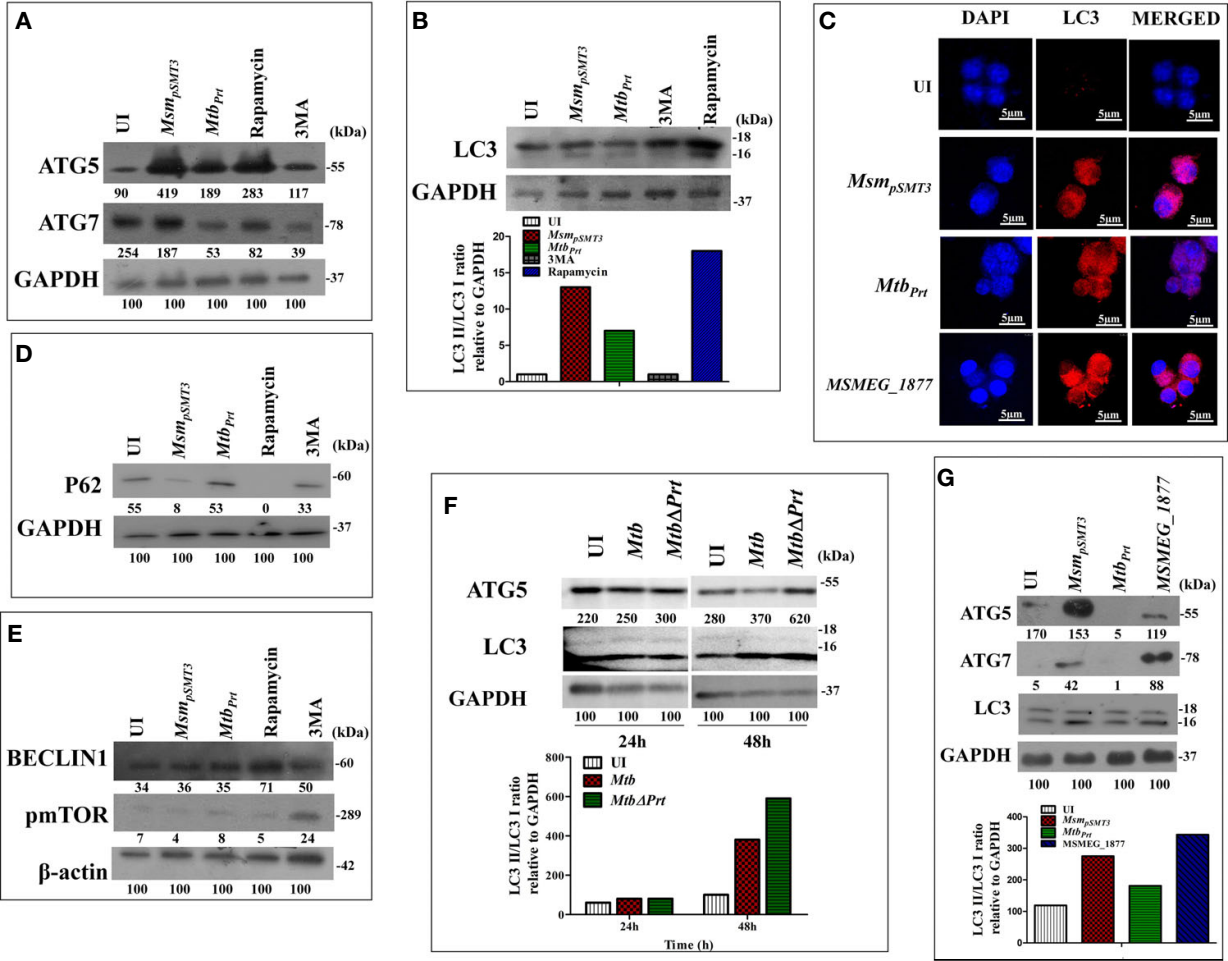
Expression of Autophagy related genes in *Msm_pSMT3_, Mtb_Prt_, Mtb*H37Rv, *Mtb*Δ*Prt* and *MSMEG_1877* infected macrophages. RAW264.7 cells were infected with *Msm_pSMT3_*and *Mtb_Prt_* for 24 h. The level of **(A)** ATG5 and ATG7 expression at protein level was checked by western blotting. The conversion of LC3I to II was estimated using **(B)** western blotting (Densitometry is representative to the particular western blot data) and **(C)** confocal microscopy using LC3I/II specific antibodies. The level of **(D)** P62**, (E)** Beclin1 and phospho- mTOR expression at protein level was checked by western blotting in RAW264.7 infected with *Msm_pSMT3_*and *Mtb_Prt_*. **(F)** The level of ATG5 and conversion of LC3-I to II was checked in *Mtb* H37Rv and *Mtb* Δ*Prt* infected RAW264.7 cells by western blotting after 48 h of infection. **(G)** The conversion of LC3I to II, ATG5 and ATG7 expression in RAW264.7 infected with *Msm_pSMT3_, Mtb_Prt_* and *MSMEG_1877* was checked by western blotting. The experiments were performed in triplicate (n=3). *Msm_pSMT3-_ Msm* harbouring pSMT3 plasmid; *Mtb_Prt_*- recombinant *Msm* expressing *MtbPrt (Rv3242c)*; *MtbΔPrt –Mtb* Prt deletion mutant in *Mtb* H37Rv.

*Mtb* inhibits autophagy to increase its intracellular persistence (Deretic et al., 2006; Chandra et al., 2015). Our recent study showed that *Mtb* PRT, encoded by *Rv3242c*, promotes mycobacterial survival in macrophages and zebrafish by inhibiting autophagy (Mohanty et al., 2015). Based on these observations, we first determined the expression of various autophagy markers such as LC3I/II, Atg5, Atg7, Beclin-1 and sequestosome 1 p62/SQSTM1 in uninfected and *Mtb* PRT-exposed macrophages. For this, we used two models. First, wild-type *Mtb* PRT was episomally expressed in *Mycobacterium smegmatis* (*Mtb_Prt_*), and we also constructed *Mtb* PRT deletion mutant (*MtbΔPrt*). *Mycobacterium smegmatis* (*Msm)* is an established surrogate model organism for the study of *Mtb* virulence proteins (Mohanty et al., 2015; Yaseen et al., 2015; Mohanty et al., 2016; Padhi et al., 2016; Sethi et al., 2016; Padhi et al., 2019). *Mtb_Prt_ -*exposed macrophages had lower expression of autophagic markers such as Atg-5 and Atg-7 (**Figure 1A**), which are recruited to the phagosomal compartments during autophagic vesicle formation. Moreover, the conversion of LC3-I to the characteristic autophagic induction marker LC3-II (**Figure 1B**) was lower than *Msm_pSMT3_*-infected (vector control) and uninfected macrophages. Autophagy is also characterized by the distribution of LC3 protein as puncta in the cytoplasm. Confocal microscopy showed LC3 puncta were less-widely distributed in *Mtb_Prt_*-infected macrophages (**Figure 1C**). We also examined the expression of the autophagic flux marker p62 (SQSTM1). During autophagy induction, p62/SQSTM1 binds to LC3 and is subsequently degraded, however when autophagy is inhibited, p62 accumulates (Pankiv et al., 2007). As shown in **Figure 1D**, p62/SQSTM1 accumulated in *Mtb_Prt_*-infected macrophages but not in *Msm_pSMT3_*-infected cells. In accordance with our previous report (Mohanty et al., 2015), *Mtb_Prt_* did not significantly alter the expression of Beclin1 (**Figure 1E**). As anticipated, treatment with an autophagy inducer (rapamycin, 50 nM) or inhibitor (3-methyladenine, 10mM) significantly induced or inhibited Atg-5, Atg-7, LC3-II and p62 proteins, respectively. We did not observe any measurable differences in the level of in phospho-mTOR (p-mTOR), an autophagy regulator, in *Mtb_Prt_*-exposed and control macrophages (**Figure 1E**). These results indicate that *Mtb* PRT inhibits autophagy through an mTOR-independent mechanism.

To confirm the role of *Mtb* PRT in autophagy inhibition, we compared the expression of Atg-5 and LC3-II in *Mtb* H37Rv- (wild-type) and *MtbΔPrt* mutant-infected macrophages. Macrophages infected with *Mtb ΔPrt* (**Figure S1**) had higher expression of Atg-5 and LC3-II (**Figure 1F**) than *Mtb* H37Rv-infected macrophages.

Comparative genomic analysis showed that the *Msm* genome contains MSMEG_1877, an orthologue of *Mtb* PRT. To preclude an effect of MSMEG_1877 in autophagy inhibition, we checked the expression of LC3-I/II, Atg-5 and Atg-7 in macrophages infected with an *Msm* strain that over-expressed *MSMEG_1877*. Autophagy was not inhibited in these macrophages (**Figure 1G**), indicating that only *Mtb* PRT, and not MSMEG_1877, is involved in autophagy inhibition. Here, and in our previous studies, we found that the *Mtb* PRT deletion mutant (*MtbΔPrt*) did not inhibit autophagy, whereas *Mtb* PRT inhibit autophagy, and so we selected the *Mtb_Prt_* strain (unless otherwise mentioned) for further experiments.

### *M. tuberculosis* Phosphoribosyltransferase Induces Histone Hypermethylation in Macrophages

**Figure 2:**
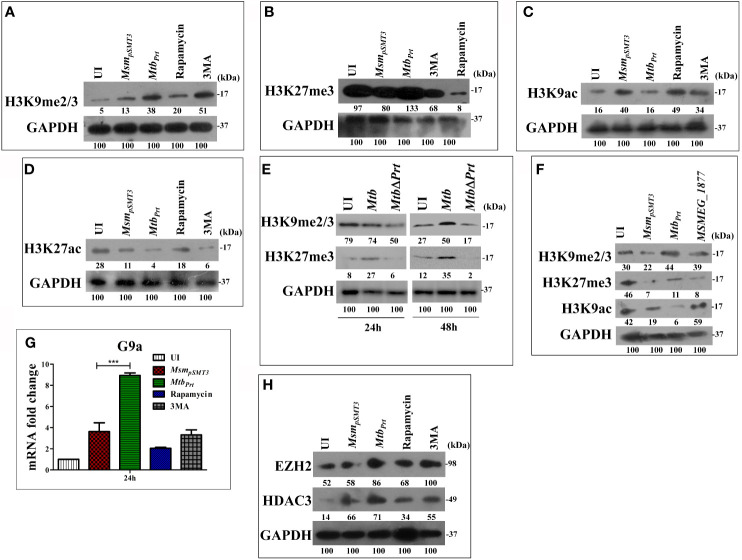
Expression of Histone hypermethylation and acetylation in RAW macrophages infected with *Msm_pSMT3_, Mtb_Prt_*, *Mtb* H37Rv, *Mtb* Δ*Prt* and *MSMEG_1877.* RAW264.7 cells were infected with *Msm_pSMT3_*and *Mtb_Prt_*for 24 h. Additionally, RAW 264.7 cells were treated with rapamycin and 3MA for 2 h. The expression of **(A)** H3K9me2/3, **(B)** H3K27me3, **(C)** H3K9ac and **(D)** H3K27ac was checked at protein level by western blotting using specific antibodies. **(E)** The level of H3K9me2/3 and H3K27me3 was checked in *Mtb* H37Rv and *Mtb* Δ*Prt* infected RAW264.7 cells by western blotting after 24 and 48 h of infection. **(F)** The expression of H3K9me2/3, H3K27me3 and H3K9ac in RAW264.7 infected with *Msm_pSMT3_*, *Mtb_Prt_*and *MSMEG_1877* was checked by western blotting. **(G)** The expression of G9a was checked at transcription level by qRT-PCR in macrophages infected with *Msm_pSMT3_*and *Mtb_Prt_*for 24 h. **(H)** The level of EZH2 and HDAC3 was checked by western blotting in macrophages infected with *Msm_pSMT3_* and *Mtb_Prt_*for 24 h. For qRT-PCR, *GAPDH was* taken as an internal control. The experiments were performed in triplicate (n=3). Results are shown as mean ± S.D. (error bars); ***p ≤ 0.001. *Msm_pSMT3-_ Msm* harbouring pSMT3 plasmid; *Mtb_Prt_*- recombinant *Msm* expressing *Mtb Prt (Rv3242c)*; *MtbΔPrt –Mtb* Prt deletion mutant in *Mtb* H37Rv.

Several pathogenic bacteria such as *S. flexneri, L. monocytogenes, Helicobacter pylori* and *Mtb* induce histone modifications to alter host immune responses in favour of a pathogen (Hamon et al., 2007; Hamon and Cossart, 2008; Ohsumi, 2014). Few epigenetic modifications regulate autophagy under non-infectious disease conditions (Artal-Martinez de Narvajas et al., 2013; Füllgrabe et al., 2014; Baek and Kim, 2017). H3K9me2/3 and H3K27me3 are histone modifications that predominantly repress transcription (Bannister et al., 2001; Ngollo et al., 2017). Therefore, we hypothesised that *Mtb* PRT may inhibit autophagy by introducing histone modifications that repress transcription of autophagy-related genes. Indeed, our western blot analysis showed that the levels of H3K9me2/3 (**Figure 2A**) and H3K27me3 (**Figure 2B**) were significantly higher in *Mtb_Prt_*(*M. smegmatis* harbouring *Mtb PRT*) infected macrophages than in *Msm_pSMT3_*(*M. smegmatis* harbouring only pSMT3 vector)-infected cells, suggesting that these modifications play a role in regulating autophagy. Rapamycin treatment down-regulated H3K9me2/3 and H3K27me3 levels, whereas 3MA treatment increased these histone modifications (**Figures 2A, B**).

Few bacterial proteins are able to concomitantly induce different histone modifications to change the dynamics of host gene expression to favour pathogen survival (Pennini et al., 2006; Hamon et al., 2007; Kumar et al., 2012; Yaseen et al., 2015). As detailed above, transcription was repressed in *Mtb* PRT-infected macrophages. Next, we examined the effect of *Mtb* PRT on the activation of transcription, *i.e.*, H3K9 and H3K27 acetylation modifications. We observed that the levels of H3K9ac (**Figure 2C**) and H3K27ac (**Figure 2D**) were significantly lower in *Mtb_Prt_*-infected cells than in *Msm_pSMT3_*-infected macrophages, suggesting that *Mtb* PRT can induce dual histone modifications, *i.e.*, histone hypermethylation and histone deacetylation. Further, the levels of H3K9me2/3 and H3K27me3 did not change in *Mtb ΔPrt*-infected macrophages (**Figure 2E**). We confirmed that the *Mtb* PRT orthologue MSMEG_1877 does not affect these histone modifications. The expression levels of H3K9me2/3, H3K27me3 and H3K9ac (**Figure 2F**) were significantly lower in *Msm_pSMT3_*- and *MSMEG_1877*-infected macrophages than in *Mtb_Prt_*-infected cells. These results suggest that *Mtb* PRT, but not MSMEG_1877, induces histone modifications that repress transcription.

### Induction of Histone Hypermethylation and Histone Deacetylation Is Mediated Through EHMT2/G9a Methyltransferase and Histone Deacetylase 3

Several histone methyltransferases such as Eset, KMT1E, G9a/EHMT2, Suv38H1 and EZH2 are responsible for histone hypermethylation. G9a (also known as euchromatin histone-lysine N-methyltransferase2, EHMT2) is a key histone methyltransferase that methylates H3K9. EZH2, a catalytic subunit of polycomb repressive complex 2 (PRC2), is another highly conserved histone methyltransferase that hypermethylates H3K27 (Fritsch et al., 2010). Next, we aimed to identify the specific methyltransferases responsible for *Mtb* PRT-induced histone hypermethylation. The expression of G9a (**Figure 2G**; P ≤ 0.001) and EZH2 (**Figure 2H**) methyltransferases were significantly higher in *Mtb_Prt_*-infected macrophages.

Next, we attempted to identify the histone deacetylase enzyme that reduces H3K9 and H3K27 acetylation in *Mtb_Prt_*-infected cells. Histone deacetylation is catalysed by various histone deacetylases (HDAC) such as HDAC1, HDAC2 and HDAC3 (Seto and Yoshida, 2014). Specifically, H3K9 and H3K27 deacetylation is induced by HDAC1, HDAC2 and HDAC3 (Večeřa et al., 2018; Præstholm et al., 2020; Gandhi et al., 2021).We observed that the level of HDAC3 was higher in *Mtb_Prt_*-infected macrophages than in control cells (**Figure 2H**), whereas HDAC1 and HDAC2 expression levels did not change (**Figure S2**). These data show that *Mtb* PRT-induced HDAC3 expression mediates H3K9 and H3K27 deacetylation.

### H3K9 Hypermethylation at the Atg5 and Atg7 Promoters *via* G9a Methyltransferase Activity Mediates Autophagy Inhibition

**Figure 3:**
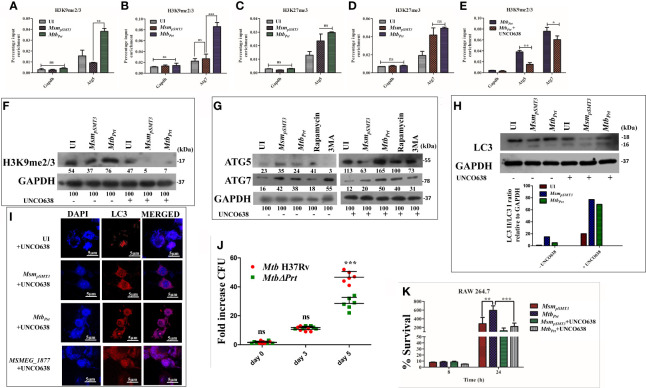
Role of H3K9 and H3K27 hypermethylation in inhibition of autophagy by *Mtb_Prt_*.. ChIP assay was performed to check the H3K9me2/3 enrichment at **(A)** *Atg5* and **(B)** *Atg7* promoter after infecting RAW264.7 with *Msm_pSMT3_*and *Mtb_Prt_*. To check the H3K27me3 enrichment at **(C)** *Atg5* and **(D)** *Atg7*, ChIP assay was performed after infecting RAW macrophages with *Msm_pSMT3_*and *Mtb_Prt_*. **(E)** ChIP assay was performed to check the H3K9me2/3 enrichment after treatment with G9a inhibitor. Quantification of the data was done by qRT-PCR using gene specific ChIP primers. RAW264.7 cells were infected with *Msm_pSMT3_*and *Mtb_Prt_* followed by treatment with UNC0638 (G9a inhibitor) for 24 h. Expressions of **(F)** H3K9me2/3, **(G)** ATG5 and ATG7, and **(H)** LC3-I to II conversion were checked by western blotting after 24 h of infection (Densitometry is representative to the particular western blot data). **(I)** LC3 puncta formation was confirmed by performing confocal microscopy in cells infected with *Msm_pSMT3_*, Mtb_Prt_ and *MSMEG_1877* followed by treatment with UNCO638 treatment for 24 h. **(J)** RAW264.7 were infected with *Mtb* H37Rv and *Mtb* Δ*Prt* strains. Cells were lysed and intracellular survival was determined 0, 3- and 5-days post-infection by a CFU assay. **(K)** RAW 264.7 were infected with *Msm_pSMT3_*and *Mtb_Prt_*strains followed by UNCO638 treatment. Cells were lysed and intracellular bacterial survival was determined 8 and 24 h post-infection by a CFU assay. Experiments were performed in triplicate (n = 3). Results are shown as mean ± S.D. ***p ≤ 0.001; **p ≤ 0.01; *p ≤ 0.05.; ns, not significant. *Msm_pSMT3-_ Msm* harbouring pSMT3 plasmid; *Mtb_Prt_*- recombinant *Msm* expressing *Mtb Prt (Rv3242c)*; *MtbΔPrt –Mtb* Prt deletion mutant in *Mtb* H37Rv.

Since we observed autophagy inhibition and increase in the levels of H3K9me2/3 and H3K27me3 modifications in *Mtb_Prt_*-infected macrophages, we performed ChIP-qPCR assay to enumerate the enrichment of these two histone hypermethylation modifications at the promoter region of autophagy-related genes. H3K9me2/3 was significantly enriched at the promoter regions of both *Atg5* (**Figure 3A**; P ≤ 0.01) and *Atg7* (**Figure 3B**;P ≤ 0.001) in response to *Mtb_Prt_* bacterial infection, while no such enrichment was observed in uninfected and *Msm_pSMT3_*-infected cells. However, H3K27me3 was not enriched at either *Atg5* (**Figure 3C**) or *Atg7* (**Figure 3D**) promoters under similar infection conditions. These results suggest that *Mtb* PRT inhibits autophagy by promoting H3K9me2/3 at the *Atg5* and*Atg7* promoters. As *Mtb* PRT did not alter Beclin1 expression, we did not investigate H3K9me2/3 enrichment at the Beclin1 promoter.

To investigate if the H3K9me2/3 enrichment at the *Atg5* and *Atg7*promoters is dependent on G9a, we used the G9a inhibitor UNCO638 (5µM). Results of ChIP-qPCR analysis showed that inhibition of G9a significantly reduced the enrichment of H3K9me2/3 at the *Atg5* and *Atg7* promoters in *Mtb_Prt_*-infected cells (**Figure 3E**; P ≤ 0.01, P ≤ 0.05), thus strongly supporting the role of G9a in H3K9me2/3 enrichment at the *Atg5* and *Atg7* promoters. We did not perform a ChIP-qPCR assay with an EZH2 inhibitor due to absence of H3K27me3 enrichment at either the *Atg5* or *Atg7* promoters.

### Inhibition of G9a Methyltransferase Abrogates H3K9me2/3- Mediated Autophagy Inhibition

Because our above results established that *Mtb* PRT induces H3K9me2/3 at the *Atg5* and *Atg7* promoters, we further investigated the role of G9a mediated-H3K9me2/3 in autophagy inhibition. Immunoblot analysis showed that G9a inhibitor (UNCO638) treatment abrogated the induction of H3K9me2/3 after *Mtb_Prt_*-infection (**Figure 3F**). Next, we investigated the effect of UNCO638 on the expression of autophagy-related proteins. We found that G9a inhibition reversed the down-regulation of ATG5 and ATG7 (**Figure 3G**) and LC3 (**Figure 3H**) by *Mtb* PRT. Confocal microscopy analysis showed that UNCO638 significantly increased in the number of LC3 puncta in *Mtb_Prt_*-infected cells (**Figure 3I**). These results clearly indicate that the inhibition of autophagy by *Mtb*Prt was due to G9a-dependent H3K9me2/3 hypermethylation.

### Down-Regulation of H3K9me2/3 Augments Clearance of Mtb_Prt_

Our previous results indicated that the presence of *Mtb* PRT inside macrophages inhibits autophagy to promote mycobacterial survival (Mohanty et al., 2015). In contrast, deletion of *Mycobacterium marinum mimG* (*MmΔmimG*), an orthologue of *Mtb* PRT, decreases bacterial survival and TB pathology in zebrafish (Mohanty et al., 2015). In the present study we also found that the deletion of *Mtb* PRT reduced the survival of the *MtbΔPrt* mutant in macrophages (at day 5) compared with wild-type *Mtb* (**Figure 3J**; P ≤ 0.001). Next, we assessed the impact of H3K9 hypermethylation and autophagy inhibition on the intracellular survival of *Mtb*. We observed that, in contrast to the untreated cells, inhibition of G9a decreased the survival of intracellular *Mtb_Prt_* 24 h after infection (**Figure 3K**; P ≤ 0.001); inhibition of autophagy further increased the survival of *Mtb_prt_* (**Figure S3**). Altogether these findings strongly suggest that *Mtb* PRT promotes bacterial survival by inhibiting autophagy through histone hypermethylation.

### *M. tuberculosis* Phosphoribosyltransferase Induces H3K9 Hypermethylation Followed by Autophagy Inhibition Is Dependent on the p38-MAPK Signalling Pathway

**Figure 4:**
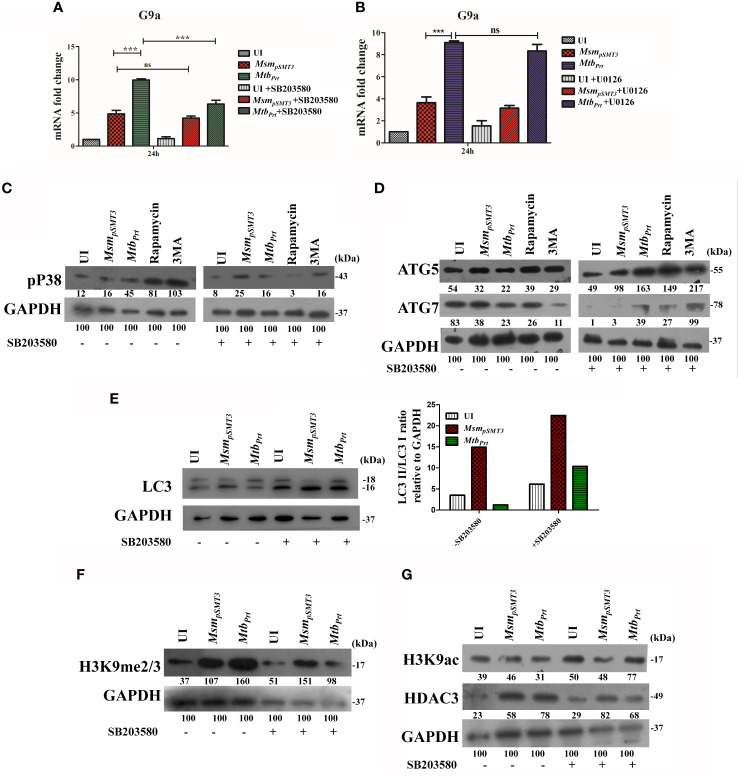
Role of MAPK in H3K9 hypermethylation and autophagy inhibition. Expression of G9a transcripts was checked in RAW264.7 cells infected with *Msm_pSMT3_*and *Mtb_Prt_*followed by treatment with **(A)** SB203580 and **(B)** U0126 for 24 h. Expression of **(C)** p-P38, **(D)** ATG5 and ATG7, **(E)** LC3-I to II conversion (Densitometry is representative to the particular western blot data), **(F)** H3K9me2/3, **(G)** H3K9ac and HDAC3 were checked in RAW cells infected with *Msm_pSMT3_*and *Mtb_Prt_* in presence and absence of SB203580 (P38 inhibitor) for 24 h. The experiments were performed in triplicate (n=3). *Msm_pSMT3-_ Msm* harbouring pSMT3 plasmid; *Mtb_Prt_*- recombinant *Msm* expressing *MtbPrt (Rv3242c)*. Results are shown as mean ± S.D. ***p < 0.001; ns, not significant.

MAPK pathways regulate eukaryotic gene expression by inducing epigenetic modifications (Vermeulen et al., 2009). Previously, we showed that *Mtb* PRT activates p-ERK and p38-MAPK signalling pathways (Mohanty et al., 2015). In this context, we investigated if MAPK signalling cascades regulate H3K9me2/3 and autophagy. First, we evaluated the level of G9a transcripts in the presence and absence of ERK (U0126, 10µM) and p38 (SB203580, 10µM) inhibitors. The p38 inhibitor decreased G9a expression in *Mtb_Prt_*-infected cells (**Figure 4A**; P ≤ 0.001) but the ERK inhibitor did not decrease G9a expression (**Figure 4B**). This result suggests that p38-MAPK plays a role in H3K9me2/3 modification and autophagy inhibition. We confirmed that treatment with SB203580 inhibitor abated the induction of p38 by *Mtb_Prt_*(**Figure 4C**). Next, we assessed the expression of ATG5, ATG7 and LC3I/II in the presence and absence of the p38 inhibitor. SB203580 significantly inhibited expression levels of ATG5 (**Figure 4D**), ATG7 (**Figure 4D**) and LC3I/II (**Figure 4E**) in *Mtb_Prt_*-infected cells. These results confirm that the inhibition of autophagy by *Mtb* PRT induced H3K9 hypermethylation is dependent on the p38-MAPK signalling pathway. Finally, we investigated the effect of SB203580 on the expression of H3K9me2/3, H3K9ac and HDAC3. The immunoblot analysis demonstrated that p38 inhibition decreased the expression of H3K9me2/3 (**Figure 4F**) and increased the level of H3K9ac (**Figure 4G**) in *Mtb_Prt_*-infected macrophages. An increase in H3K9ac could be attributed to reduced expression of HDAC3 due to inhibition of p38 expression (**Figure 4G**). These results suggest that *Mtb* PRT-mediated H3K9 hypermethylation followed by autophagy inhibition is facilitated by activation of the p38-MAPK signalling pathway.

### *M. tuberculosis* Phosphoribosyltransferase Induces Histone Hypermethylation and Inhibits Autophagy in Murine Bone Marrow-Derived Macrophages

**Figure 5:**
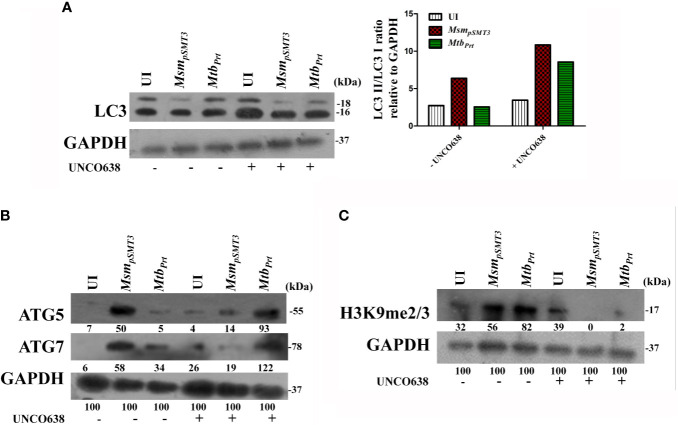
Expression of histone hypermethylation and autophagy in bone marrow derived macrophages infected with *Msm_pSMT3_* and *Mtb_Prt_.* Bone marrow derived macrophages were infected with *Msm_pSMT3_* and *Mtb_Prt_*strains followed by treatment with UNCO638 (G9a inhibitor) for 24 h. Western blot analysis was performed to check the **(A)** conversion of LC3I to II (Densitometry is representative to the particular western blot data), and expression of **(B)** ATG5 and ATG7, and **(C)** H3K9me2/3. The experiments were performed in triplicate (n=3). *Msm_pSMT3-_ Msm* harbouring pSMT3 plasmid; *Mtb_Prt_*- recombinant *Msm* expressing *MtbPrt (Rv3242c)*.

To confirm our key findings from a murine cell line (RAW264.7 macrophages), we performed representative experiments in primary bone marrow-derived macrophages (BMDM) isolated from Balb/C mice. The expression of LC3-II (**Figure 5A**), ATG5 and ATG7 (**Figure 5B**) was lower in *Mtb_Prt_*-infected BMDM than in uninfected cells, and inhibition of G9a increased the expression of these autophagic proteins. Similarly, H3K9me2/3 (**Figure 5C**) was higher in *Mtb_Prt_*-infected BMDM than in uninfected cells, and this effect was reversed by inhibition of G9a methyltransferase. Thus, similar data obtained in BMDM and RAW264.7 macrophages confirmed that *Mtb* PRT induces epigenetic modifications to inhibit autophagy and augment *Mtb* persistence in macrophages.

## Discussion

**Figure 6:**
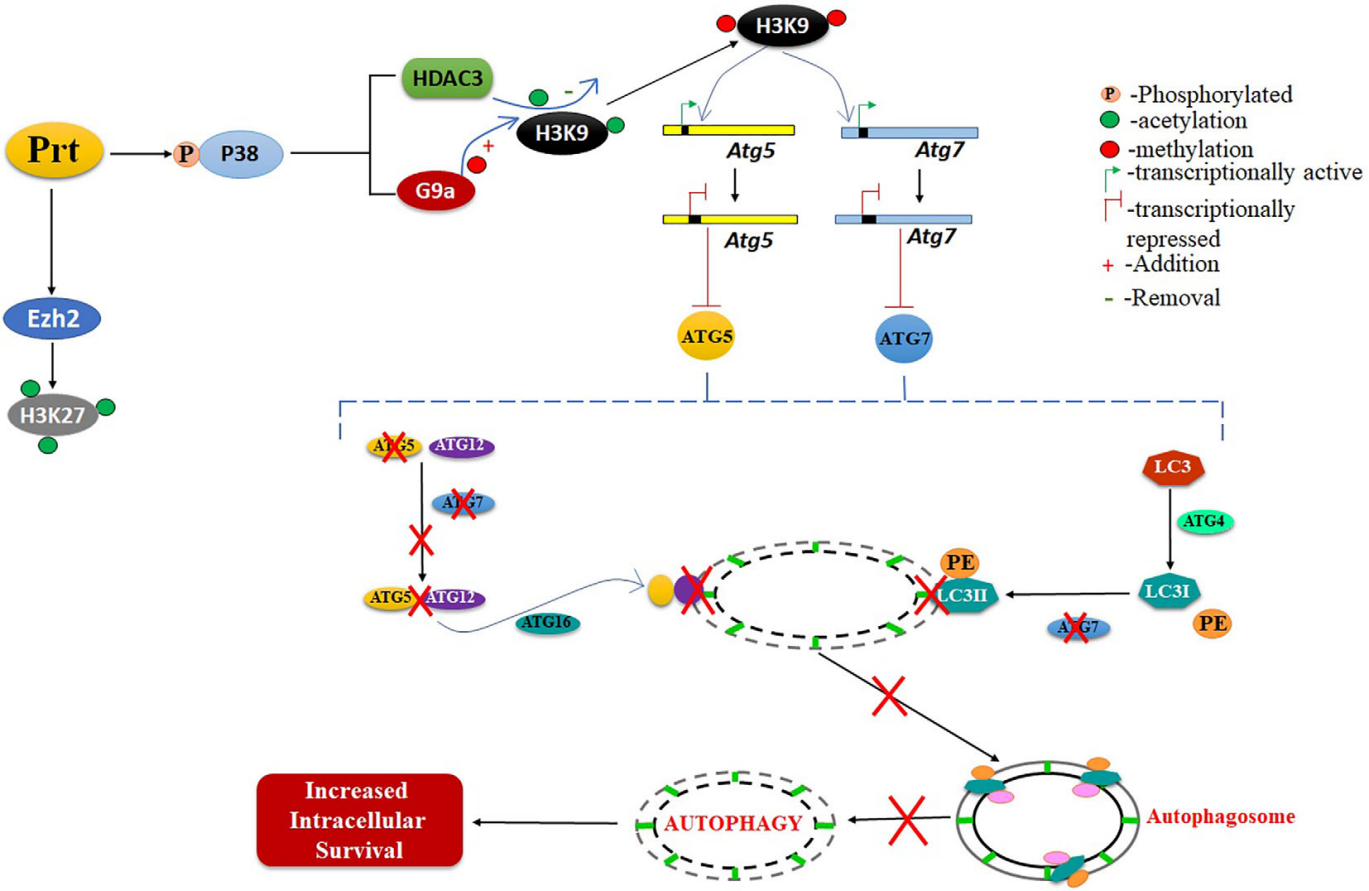
Schematic representation of role of *Mtb*PRT in induction of histone hypermethylation, which down regulates the autophagy. This downregulation of autophagy leads to increased intracellular survival.

*Mtb* employs various strategies to evade host immune responses. One mechanism involves reprogramming of host genes to modulate autophagy, thereby avoiding killing by host cells (Deretic, 2014). However, the molecular mechanisms that underlie autophagy inhibition by *Mtb* are poorly understood. Here, we report that *Mtb* PRT inhibits autophagy through an mTOR independent mechanism to promote mycobacterial persistence inside the macrophages (**Figure 6**).

*Atg5* is an autophagy-related gene that is crucially involved in *Mtb-*mediated autophagy inhibition. We found that the presence of *Mtb* PRT in macrophages significantly down-regulated the expression of LC3-II, ATG5 and ATG7. These molecules are involved in the formation of the ATG5–ATG12 complex, which is responsible for the elongation and closure of autophagosomes, generation of lipidated forms of LC3 and their localisation to the autophagosome membrane (Mizushima et al., 2011; Ohsumi, 2014; Arakawa et al., 2017; Nishimura and Tooze, 2020). In agreement with our previous report, we observed that *Mtb* PRT did not alter the expression of Beclin1, which after dissociation from the apoptosis regulator Bcl2 forms a complex with hVps34. This complex is important for the crosstalk between autophagy and apoptosis (Liang et al., 2006). Thus, the absence of any effect of *Mtb* PRT on beclin1 suggests that *Mtb* PRT is involved in autophagy but not apoptosis. To understand the underlying molecular mechanism(s) of *Mtb* infection, we investigated how epigenetic modifications contribute to *Mtb* PRT-mediated inhibition of autophagy. We found that *Mtb* infection increased H3K9, H3K27 hypermethylation (involved in transcription repression), and reduced H3K9 and H3K27 acetylation (involved in transcription activation). These results suggest that *Mtb* PRT performs dual histone modifications to favour *Mtb* survival. Histone hypermethylation is catalysed by histone methyltransferases such as G9a, Suv39h1/h2 (which catalyses H3K9 hypermethylation) and Ezh2 (which catalyses H3K27 hypermethylation) (Mozzetta et al., 2015). Our results indicate that G9a and EZH2 are involved in the induction of H3K9 and H3K27 hypermethylation in infected macrophages. On the other hand, *Mtb* PRT caused a significant decrease in H3K9ac and H3K27ac in exposed macrophages. Histone deacetylation is catalysed by histone deacetylases (HDACs) such as HDAC1, HDAC2, HDAC3 and sirtuans (Seto and Yoshida, 2014). We found that HDAC3 is predominantly responsible for deacetylation during *Mtb_Prt_* infection. Together, these results suggested that *Mtb* PRT induced histone hypermethylation and deacetylation events are responsible for the alteration of autophagy. The concurrent induction of histone hypermethylation and deacetylation has been shown in previous reports during chromosome condensation and cell cycle progression (Park et al., 2011). The levels of H3K9 and H2K27 hypermethylation and H3K9 and H3K27 deacetylation did not change inmacrophages treated with rapamycin (an autophagy inducer) or 3MA (an autophagy inhibitor), indicating that both hypermethylation and deacetylation are upstream of autophagy and are specific to *Mtb* PRT.

Hypermethylation of lysine residues on histone proteins leads to the formation of condensed chromatin which represses the transcription by preventing the binding of transcription factors (Park et al., 2011; Mozzetta et al., 2015). Thus, H3K9me2/3 or H3K27me3 enrichment at the promoter regions of target genes will inactivate transcription. Our ChIP-qPCR assay showed that H3K9me2/3, but not H3K27me3, increased at the promoters of *Atg5* and *Atg7*genes in macrophages expressing *Mtb* PRT. This finding indicates that H3K9me2/3 predominantly mediates repression of *Atg5* and *Atg7* genes, while H3K27me3 may be involved in the repression of genes other than *Atg5* and *Atg7*. Overall, our results demonstrate that *Mtb* PRT inhibits autophagy by specifically recruiting H3K9me3 at the *Atg5* and *Atg7* promoters.

We showed that *Mtb* PRT induces H3K9me2/3 by up-regulating G9a methyltransferase. Chemical inhibition of G9a decreased H3K9me2/3 expression and at the same time increased in the expression of LC3-II, ATG5 and ATG7, thus confirming that G9a methyltransferase-induced H3K9 hypermethylation is responsible for autophagy inhibition. Non-pathogenic mycobacteria such as *Msm* are readily killed by macrophages, whereas pathogenic *Mtb* survive inside macrophages (Rahman et al., 2014). Our previous report showed that episomal expression of *Mtb* PRT in non-pathogenic *Msm* increased bacterial survival in macrophages (Mohanty et al., 2015). Here, we demonstrated that deletion of *MtbPrt* (*MtbδPrt)* reduced the survival of bacteria in macrophages. These results suggest that *Mtb* PRT is a virulence factor important for *Mtb* survival. Inhibition of G9a, which demethylates H3K9, decreased the intracellular survival of *Mtb* PRT. Conversely, infection with the *MtbδPrt* mutant reduced H3K9 hypermethylation and increased H3K9ac and autophagy. Thus, our previous and present results indicate that *Mtb* PRT induces H3K9 hypermethylation by upregulating G9a methyltransferase, which inhibits autophagy, and inhibition of autophagy subsequently promotes intracellular bacterial survival.

The MAPK signaling pathway plays a crucial role in mycobacterial infection (Pasquinelli et al., 2013; Mohanty et al., 2016), yet only a limited number of mycobacterial proteins are known to induce epigenetic modifications in p38-MAPK-dependent pathways (Pennini et al., 2006; Vermeulen et al., 2009). We found that although *Mtb* PRT activates both p38-MAPK and ERK signalling pathways, histone hypermethylation followed by autophagy inhibition was specifically dependent on the p38-MAPK pathway. Inhibition of p38 decreased histone hypermethylation, which subsequently up-regulated ATG5, ATG7 and LC3-II expression. However, it is important to demonstrate these findings in *MtbδPrt* mutant. Moreover, the underlying mechanism responsible for P38 mediated histone methylation is poorly studied. There are couple of reports which show the involvement of NF-κB in p38 mediated histone modifications in *Shigella flexnari* and *Listeria monocytogenes* (Hamon and Cossart, 2008). *Mtb* PRT is also reported to increase NF-κB expression in our previously published report (Mohanty et al., 2015). Additionally, involvement of NF-κB in autophagy inhibition and intracellular survival is also well known (Djavaheri-Mergny et al., 2007; Bai et al., 2013; Espert et al., 2015). So, involvement of NF-κB in p38 mediated histone methylation in *Mtb* can be a possible mechanism which needs to be studied. Further, it remains to be investigated if inhibition of H3K9me2/3 has any impact on the survival of *Mtb* in tuberculosis mice model. In summary, to the best of our knowledge, this is the first report that shows *Mtb* induces H3K27me3 in the promoter region of autophagy-related genes to inhibit autophagy. Thus, *Mtb* PRT could be a potential drug target to improve TB therapy.

## Data Availability Statement

The raw data supporting the conclusions of this article will be made available by the authors, without undue reservation.

## Author Contributions

SS planned the experimental setup, performed the experiments, analysed the data and wrote the manuscript. BN analysed the experiments and provided technical assistance. MM performed experiments with Mtb and analysed the data. PS planned experimental setup with Mtb strain and provided resources. AS planned the experimental setup, data analysis, wrote the manuscript and provided all the necessary resources and support for the completion of the study. SM contributed in the design of the study and analysed data. All authors contributed to the article and approved the submitted version.

## Funding

This work was supported by grant (BT/PR23317/MED/29/1186/2017) from the Department of Biotechnology, Government of India to AS. SS is grateful to the Department of Science and Technology, Government of India for awarding DST-INSPIRE fellowship (IF150081). This study was supported by the University of Zurich, Institute of Medical Microbiology and Swiss National Science Foundation (IZK0Z3_154138/1 and 310030_197699).

## Conflict of Interest

The authors declare that the research was conducted in the absence of any commercial or financial relationships that could be construed as a potential conflict of interest.

## Publisher’s Note

All claims expressed in this article are solely those of the authors and do not necessarily represent those of their affiliated organizations, or those of the publisher, the editors and the reviewers. Any product that may be evaluated in this article, or claim that may be made by its manufacturer, is not guaranteed or endorsed by the publisher.

## Supplementary Material

Supplementary Figure 1 Expression of HDAC1 and HDAC2. RAW264.7 cells were infected with *Msm_pSMT3_*and *Mtb_Prt_* for 24 h. The level of HDAC1 and HDAC2 expression at protein level was checked by western blotting.The experiments were performed in triplicate (n=3).

Supplementary Figure 2 Intracellular survival of *Mtb* PRT after autophagy inhibition by 3MA. RAW 264.7 were infected with *Msm_pSMT3_*and *Mtb_Prt_*strains followed by 3MA treatment. Cells were lysed and intracellular bacterial survival was determined 8 and 24 h post-infection by a CFU assay. Experiments were performed in triplicate (n=3). Results are shown as mean ± S.D. ***, p ≤ 0.001; **, p ≤ 0.01; *, p ≤ 0.05.; ns, not significant.

Supplementary Figure 3 Southern blot analysis of *M. tuberculosis prt* locus. *M. tuberculosis* (1, wt) was transformed with *prt* targeting vector pMCS5-rpsL-hyg-Δ3242c, transformants were selected on 7H10 agar containing hygromycin. Putative single cross-over transformant (2, sco) was colony purified and subjected to streptomycin counter selection to obtain putative *Δprt* deletion mutant (3, Δ). Genomic DNA of the indicated strains was isolated, digested with *AgeI*, separated on an agarose gel, blotted and probed with a 128bp probe located upstream of the target gene. The band patterns confirm the predicted genotypes.

## Abbreviations

*Mtb*, *Mycobacterium tuberculosis*; *Msm*, *Mycobacterium smegmatis*; PRT, phosphoribosyltransferase; ATG, Autophagy-related proteins; LC3, Microtubule-associated proteins 1A/1B light chain 3B; MAPK, The mitogen activated protein (MAP) kinases; EHMT2, Euchromatic histone-lysine N-methyltransferase 2 also known as G9a; HDAC, Histone deacetylases; *Msm_pSMT3,_ Msm* harbouring pSMT3 plasmid; *Mtb_Prt_*, recombinant *Msm* expressing *MtbPrt (Rv3242c)*; *MtbΔPrt, Mtb*Prt deletion mutant in *Mtb* H37Rv; BMDM, Bone marrow derived macrophages; CFU, Colony forming units.
