# Supplementary figures and images for: Mycobacterium tuberculosis Phosphoribosyltransferase Promotes Bacterial Survival in Macrophages by Inducing Histone Hypermethylation in Autophagy-Related Genes
Source: Front Cell Infect Microbiol. 2021 Jul 26;11:676456. doi: 10.3389/fcimb.2021.676456 (PMC8350138; doi:10.3389/fcimb.2021.676456)

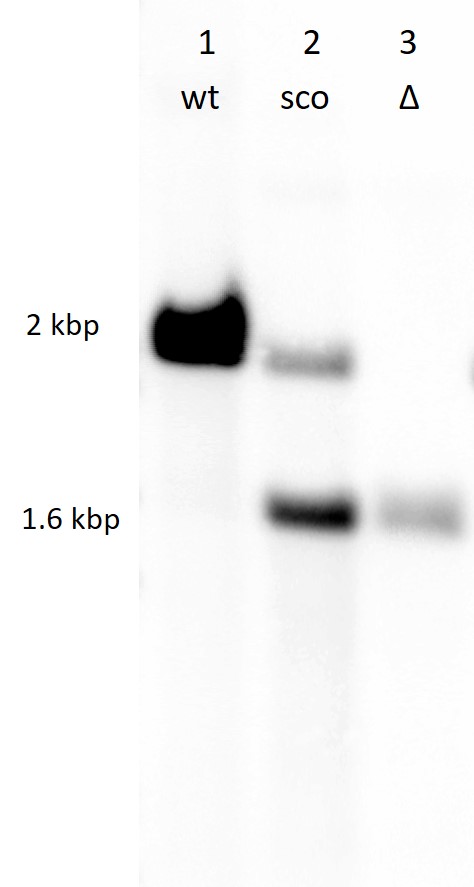

Supplement: Supplementary Figure 1 — Expression of HDAC1 and HDAC2. RAW264.7 cells were infected with MsmpSMT3and MtbPrt for 24 h. The level of HDAC1 and HDAC2 expression at protein level was checked by western blotting.The experiments were performed in triplicate (n=3). [file Image_1.jpg]

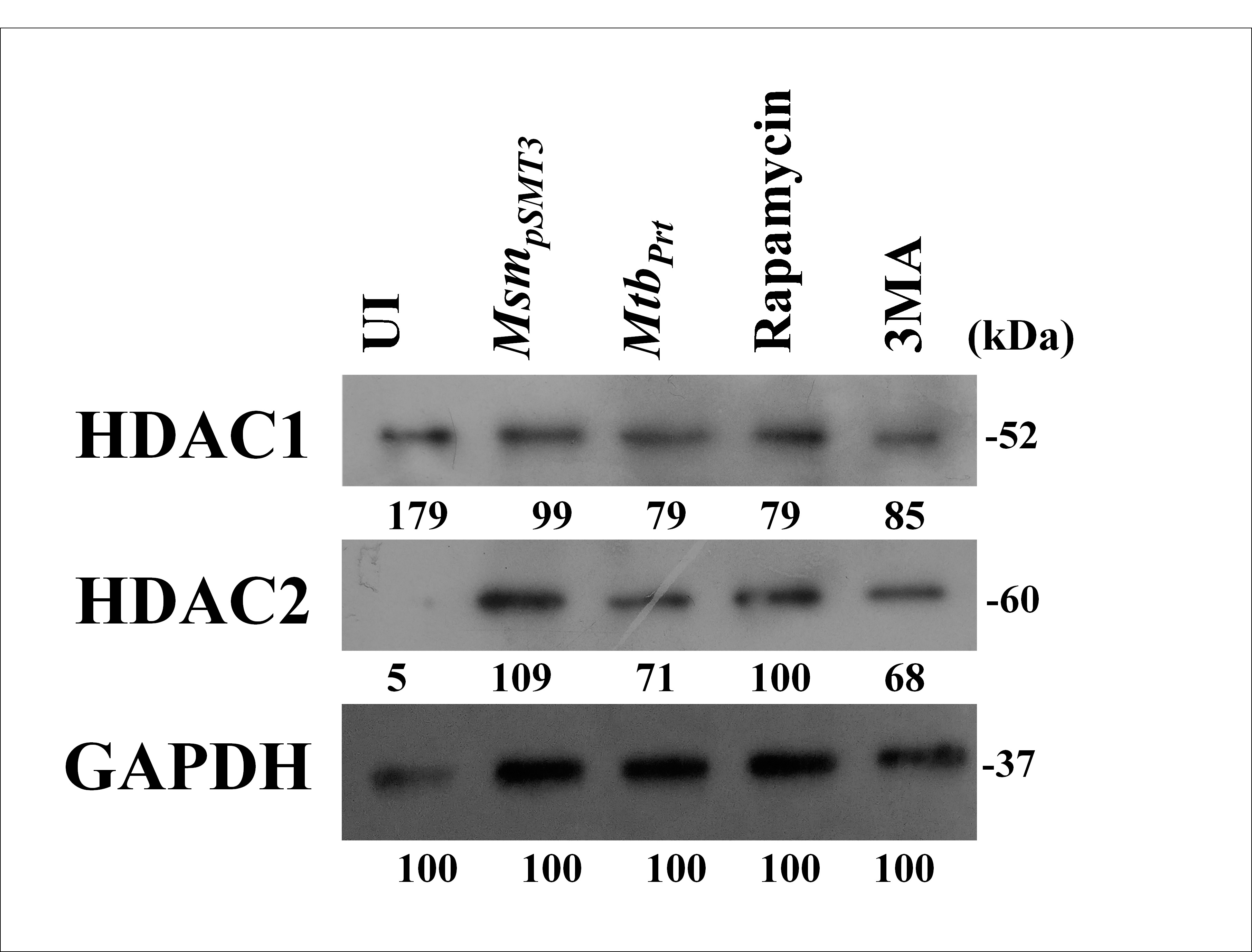

Supplement: Supplementary Figure 2 — Intracellular survival of Mtb PRT after autophagy inhibition by 3MA. RAW 264.7 were infected with MsmpSMT3and MtbPrtstrains followed by 3MA treatment. Cells were lysed and intracellular bacterial survival was determined 8 and 24 h post-infection by a CFU assay. Experiments were performed in triplicate (n=3). Results are shown as mean ± S.D. ***, p ≤ 0.001; **, p ≤ 0.01; *, p ≤ 0.05.; ns, not significant. [file Image_2.jpg]

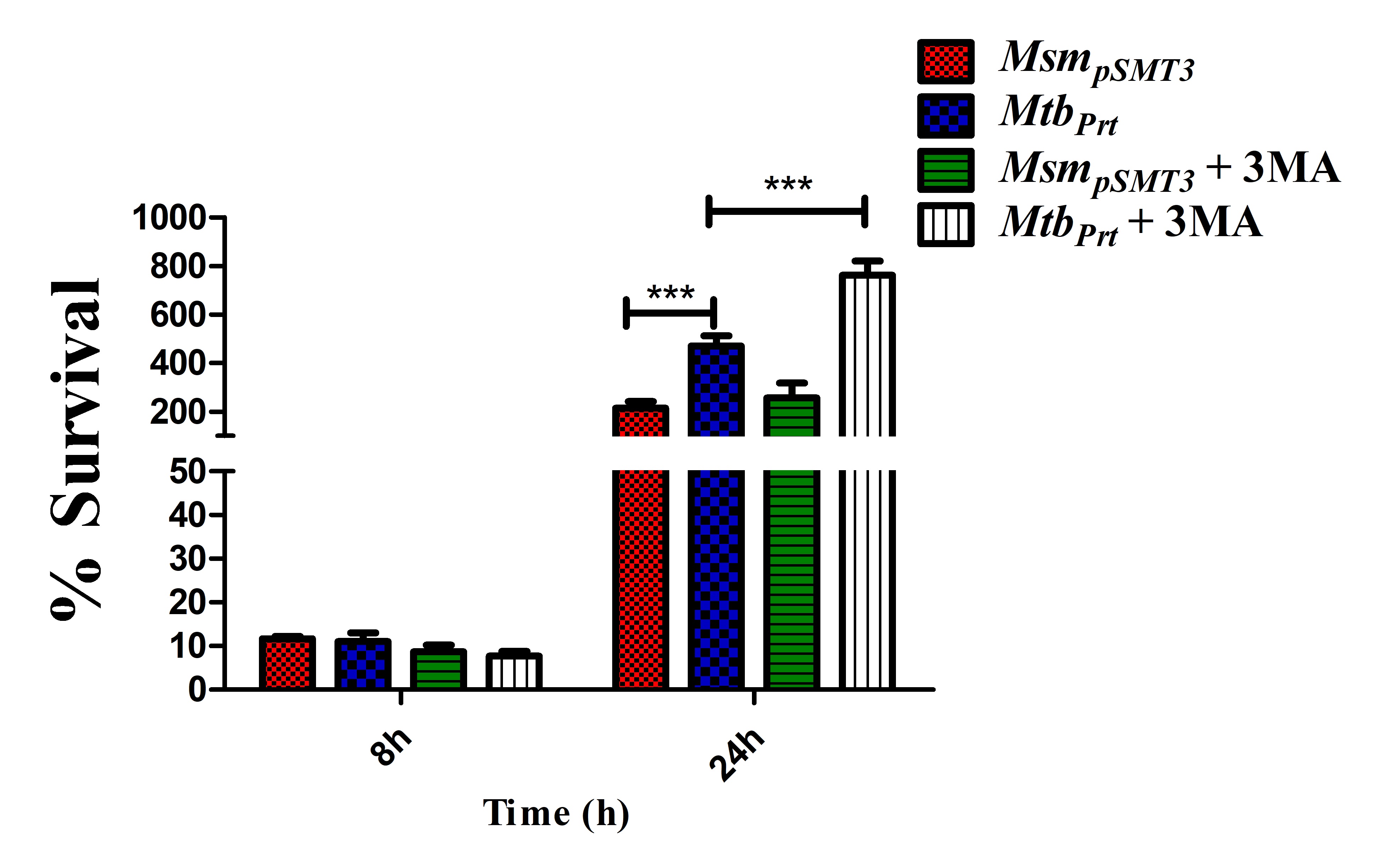

Supplement: Supplementary Figure 3 — Southern blot analysis of M. tuberculosis prt locus. M. tuberculosis (1, wt) was transformed with prt targeting vector pMCS5-rpsL-hyg-Δ3242c, transformants were selected on 7H10 agar containing hygromycin. Putative single cross-over transformant (2, sco) was colony purified and subjected to streptomycin counter selection to obtain putative Δprt deletion mutant (3, Δ). Genomic DNA of the indicated strains was isolated, digested with AgeI, separated on an agarose gel, blotted and probed with a 128bp probe located upstream of the target gene. The band patterns confirm the predicted genotypes. [file Image_3.jpg]
